# A liquid immunogenic fiducial eluter for image-guided radiotherapy

**DOI:** 10.3389/fonc.2022.1020088

**Published:** 2022-12-21

**Authors:** Michele Moreau, Geraud Richards, Sayeda Yasmin-Karim, Amol Narang, Curtiland Deville, Wilfred Ngwa

**Affiliations:** ^1^ Department of Radiation Oncology, Brigham and Women's Hospital, Dana-Farber Cancer Institute, Harvard Medical School, Boston, MA, United States; ^2^ Department of Radiation Oncology and Molecular Radiation Sciences, School of Medicine, Johns Hopkins University, Baltimore, MD, United States; ^3^ Department of Radiation Oncology & Molecular Radiation Sciences, Johns Hopkins University School of Medicine, Baltimore, MD, United States

**Keywords:** nanoparticles, smart radiotherapy biomaterials, biodegradable polymers, immunoadjuvant, radiation therapy, liquid fiducials

## Abstract

**Introduction:**

Fiducials are routinely used to provide image-guidance during radiotherapy. Here, a new nanoparticle-based liquid immunogenic fiducial is investigated for its potential to provide image-guidance, while also enhancing treatment outcomes.

**Methods:**

This fiducial, liquid immunogenic fiducial eluter (LIFE) biomaterial, is formulated with natural biodegradable polymers, chitosan and sodium alginate with radio-sensitizing nanoparticles, and immunoadjuvant like anti-CD40 monoclonal antibody. Once administered intra-tumorally, this liquid smart radiotherapy biomaterial congeals within the calcium rich tumor microenvironment. The potential use of LIFE biomaterial for providing image guidance in magnetic resonance imaging (MRI) and computed tomography (CT) was investigated over different time period in a pre-clinical tumored mouse model.

**Results:**

Results showed that the LIFE biomaterial can provide both MRI contrast and CT imaging contrast over 3-weeks, with gradual decrease of the contrast over time, as the LIFE biomaterial biodegrades. Results also showed the LIFE biomaterial significantly slowed tumor growth and prolonged mice survival (p < 0.0001) over time.

**Discussion:**

The results highlight the potential use of the LIFE biomaterial as a multi-functional smart radiotherapy biomaterial that could be developed and optimized for hypo-fractionated radiotherapy applications and combining radiotherapy with immunoadjuvants.

## 1 Introduction

In image-guided radiotherapy, inert biomaterials such as beacons or fiducials are routinely used to ensure treatment precision and accuracy, especially for tumors that may move during the treatment (1, 2). Different fiducial markers include: clips, linear gold seed, coils, or liquid fiducial markers, which have proven to be clinically safe and feasible (3–5). Liquid fiducial markers have the additional advantage of minimizing migration over time (6, 7) and minimizing any image artifacts (8, 9). Previous studies have further indicated that liquid fiducials are safer and easier to inject at the targeted site compared to solid fiducials that may require endoscopic implantation, which may be more complex for resource-limited settings and may be uncomfortable procedure for the patient (10, 11). Recent studies have highlighted the use of liquid fiducials to improve image-guided radiotherapy in different types of tumors e.g. pancreas, bladder, and prostate (11–13).

Currently-used fiducials are designed to be bio-compatible and intrinsically non-immunogenic, with no additional benefit or function than to provide image-guidance (11). However in preclinical studies, a new generation of fiducials or smart radiotherapy biomaterials (SRBs) is being developed that are immunogenic with potential to also enhance therapy outcomes and providing sustained delivery of immunoadjuvants into the tumor sub-volume during IGRT (14, 15).

In this study, we investigate a novel potentially multifunctional fiducial that is designed to be an immunogenic liquid fiducial, with capacity to provide image-guidance, and sustainably deliver immunoadjuvants to enhance therapeutic efficacy. This fiducial called a liquid immunogenic fiducial eluter (LIFE) biomaterial is composed of natural biodegradable polymers such as chitosan and sodium alginate and nanoparticles providing a versatile image-guided drug delivery system for use during radiotherapy. The objective of this paper is to investigate the potential of the LIFE biomaterial to provide CT/MR image contrast and enhance therapy outcomes *in-vivo* in animals with pancreatic tumors.

## 2 Materials and methods

### 2.1 LIFE Biomaterial fabrication

**Figure 1:**
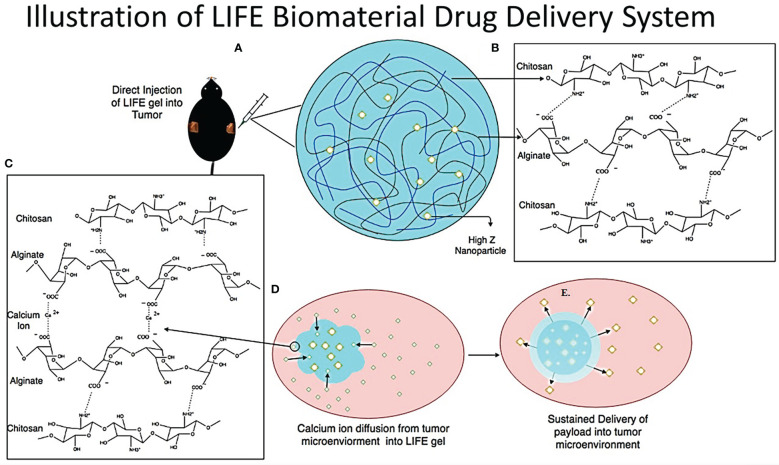
The LIFE biomaterial consists of a mixture of sodium alginate gel and chitosan gel made in a 1:1 ratio in addition to 10% of nanoparticle solution (either titanium dioxide for CT contrast or AGuIX gadolinium-based nanoparticles for MR contrast) to the polymer mixture **(B)** to enable image contrast during radiotherapy treatment. This figure highlights the molecular structure and ionic interaction between chitosan and sodium alginate polymers **(C)**. Once the LIFE biomaterial is injected in the tumor site using an insulin syringe with a (22-Gauge) needle attached **(A)**, the gel congeals within the calcium-rich tumor micro-environment as illustrated in figure 1 **(D, E)**.

The LIFE biomaterial was formulated using a mixture of natural polymers: chitosan from Deacetylated chitin, Poly (D-glucosamine), and Alginic acid sodium salt from brown algae, sodium alginate (Sigma Millipore, St. Louis, MO, USA) (**Figure 1**). A mixture of 4% (w/v) (v = 50uL) alginate biomaterial hydrogel solution added to a 1% (w/v) (v = 50uL) chitosan hydrogel solution in a 1:1 ratio, was prepared and injected in the calcium chloride (CaCl_2_) rich-tumor microenvironment. The molecular ionic interactions responsible for the jellification of the LIFE biomaterial occur between the negatively charged carboxyl groups on the sodium alginate polymer chain, and the positively charged amine groups of the chitosan polymer chain and the polyvalent cation calcium (16). The carboxyl groups on the sodium alginate chain form ionic cross linkages with the amine groups on the chitosan chain and calcium cations (17). A schematic of the *in-vivo* treatment of mice tumors with LIFE biomaterial loaded with nanoparticles (**Figure 1**) shows the hypothesized bio-distribution of the payload inside the tumor microenvironment.

### 2.2 Mouse tumor inoculation

Immunocompetent wild-type C57BL/6 strain male and female mice were acquired from Taconic Biosciences Inc. (Rensselaer, NY, USA), at 8-10 weeks old. Compliance of the animal trials followed the guidelines and regulations set by the Institutional Animal Care and Use Committee (IACUC) of the Dana Farber Cancer Institute (protocols 15-040 and 08-023). Pancreatic cancer cell line, KPC cell line derived from an LSL-Kras; p53+/floxed, Pdx-cre mouse. Ptf1/p48-Cre (KPC) cells were a gift from Dr. Anirban Maitra (MD Anderson Cancer center). KPC cells were cultured in Roswell Park Memorial Institute medium (RPMI, Life Technologies, Wal-tham, MA, USA) supplemented with 10% FBS (Thermofisher Scientific, Waltham, MA, USA), 2 mmol/L L-glutamine (Thermofisher Scientific, Waltham, MA, USA), 1% penicillin/streptomycin (Thermofisher Scientific, Waltham, MA, USA), 1% MEM non-essential amino acids (Thermofisher Scientific, Waltham, MA, USA), 1 mmol/L sodium pyruvate, and 0.1 mmol/L β-mercaptoethanol (Thermofisher Scientific, Waltham, MA, USA). All cells were cultured at 37°C in a humidified incubator with 5% CO_2_. Mice were inoculated subcutaneously with 150,000 live KPC cells/flank as determined by the Luna cell counter (Logos Biosystems, Annandale, VA, USA) using a trypan blue staining (Thermofisher Scientific, Waltham, MA, USA) for tumor formation. Growth of tumor was monitored after cancer cell inoculation until when palpable tumor growth was observed. Tumor dimensions were evaluated with the aid of a digital Vernier caliper. Tumor volume was analyzed as follows V = [1/2 *L*(W^2^)] where L and W represent the length and width of the tumor. The length was considered across the ideal longitude of the lower limb, and the width was considered along the latitude.

### 2.3 MR Imaging of LIFE biomaterial in mice pancreatic tumor

In one experiment, gadolinium-based nanoparticles, AGuIX, provided by NH Theraguix (Lyon, France) with MRI contrast were used as the nanoparticle component of the LIFE biomaterial. MRI experiments were carried out on a 7T Bruker BiosSpec superconducting magnet system (Bruker Corp, Billerica, MA, USA) with a 30 cm USR flat core. The same parameters and mouse setup was used following a previously published study (18). Similar to previous work (18), the MRI system was calibrated and used to investigate different formulations of the LIFE biomaterial as described in the LIFE biomaterial fabrication method above. C57BL6/Tac female mice (8-10 weeks old) were subcutaneously injected with pancreatic cancer (KPC) cells at a cell density of 150,000-cells/flank. The cohorts of mice investigated were (n = 1/group): a) two-injections of 2% (w/v) (v = 50 uL/injection) of sodium alginate biomaterial in addition to 6% (w/v) (v = 100 uL) of calcium chloride (CaCl_2_) (Sigma Millipore, St. Louis, MO, USA); b) One-injection of 4% (w/v) (v = 100 uL) of sodium alginate biomaterial and 6% (w/v) (v = 100 uL) of CaCl_2_; c) 2% (w/v) (v = 50 uL) of sodium alginate biomaterial mixed 1:1 ratio with 1% (w/v) (v = 50 uL) of chitosan biomaterial and 6% (w/v) (v = 100 uL) of CaCl_2_; d) 4% (w/v) (v = 50 uL) of sodium alginate biomaterial mixed 1:1 ratio with 1% (w/v) (v = 50 uL) of chitosan biomaterial and 10uL of AGuIX (c = 4.1mg/mL) nanoparticles was added to the mixture. MR scans were taken for prior to injection and post injection of the LIFE biomaterial for the following time points: 10 min, 3 hours, and day 1, 2, 4, and 7. Similarly to previous work (18), a fast spin echo (FSE) sequence was generated for these mice experiments to yield T1-weighted images for placement purposes, as well as for T1 maps. The scanning parameters for the T1-map sequence were similar to those mentioned in previous work (18).

### 2.4 CT imaging of LIFE biomaterial *in-vivo*

C57BL6/Tac male mice (6 – 8 weeks old) were subcutaneously injected with pancreatic cancer (KPC) cells at a cell density of 150,000 cells/flank on both flanks of mice. A LIFE biomaterial formulation was prepared as follows: a mixture of 4% (w/v) (v = 50uL) alginate biomaterial solution added to a 1% (w/v) (v = 50uL) chitosan solution in a 1:1 ratio, and v = 10 uL of titanium dioxide (TiO_2_) nanoparticles (c = 0.5g/mL) was added to the polymer mixture (US Research Nanomaterials, Inc., Houston, TX, USA) for CT contrast. The monoclonal antibody (mAb), Ab-CD40 (v = 2.3uL/injection), was added to the polymer mixture to investigate its effect on tumor growth and overall mice survival. The monoclonal antibody anti-mouse CD40 (FGK4.5/FGK45) was bought from BioXcell (New-Hampshire, USA). The resulting LIFE biomaterial was kept on ice before injection in mice tumors. The cohorts of mice imaged were: LIFE biomaterial loaded with TiO_2_ nanoparticles known as LIFE_TiO_2_ (n = 3); and LIFE biomaterial loaded with TiO_2_ and 20ug of anti-CD40 (Ab-CD40) monoclonal anti-body known as LIFE_TiO_2__Ab-CD40 (n = 3). CT scans were taken pre-injection of biomaterial, and subsequently at 10 min, days 1, 7, 14, and 21 post injection.

### 2.5 Investigating the therapy enhancing potential of the LIFE biomaterial loaded with Anti-CD40 monoclonal antibody

Twenty C57BL6N/TAC male mice received subcutaneous injections of 150,000 cells/flank of KPC pancreatic tumor cell line on one flank and were monitored for tumor growth for 2 weeks prior to the tumors being palpable reaching approximately (diameter > = 4) mm in size. Similar LIFE biomaterial formulations using TiO_2_ with the addition of anti-CD40 mAb as was used for the CT imaging of LIFE biomaterial study were prepared. The cohorts were as followed: a) Control received no treatment (n = 6); b) single fraction of RT at 5 Gy (n = 4); c) single intra-tumoral injection of LIFE_TiO_2__Ab-CD40 (n = 4); d) single intra-tumoral injection of LIFE_TiO_2__Ab-CD40_5Gy where one fraction of IGRT at 5 Gy was administered (n = 6). The small animal radiation research platform, SARRP (Xstrahl, Suwanee, GA, USA), was used to irradiate the cohorts of mice using 220 kVp, 13 mA, 10 × 10 collimator and 0.15 mm copper (Cu) filter at 5 Gy. Tumor volume and mice survival were assessed over 10-weeks timespan.

### 2.6 Statistical analysis

For survival study, statistical analyses was done using GraphPad prism v9.0. The Kaplan-Meier statistics (Madsen 1986, Statistical Concepts, Prentice Hall, Englewood Cliffs, NJ, USA) was employed. P-value **** p < 0.0001 was considered statistically significant.

## 3 Results

### 3.1 Assessment of LIFE biomaterial for IGRT image-contrast in mice pancreatic tumors

**Figure 2:**
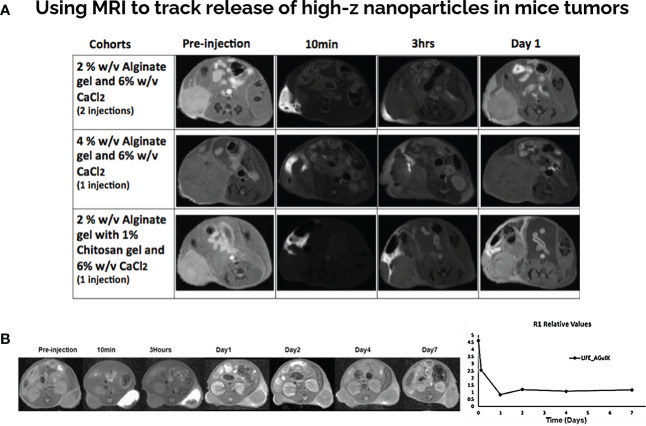
LIFE Biomaterial with gadolinium-based nanoparticles show MR contrast over time in mice pancreatic tumors. **(A)** Different formulations of LIFE gel was tested in the search of prolonging the MR signal for more than 24hrs. **(B)** LIFE biomaterial was produced using 4% (w/v) (v = 50uL) of sodium alginate biomaterial mixed 1:1 ratio with 1% (w/v) (v = 50 uL) of chitosan biomaterial. 10 uL of AGuIX (c = 4.1 mg/mL) nanoparticles was added to the mixture. MR images showed gadolinium contrast from LIFE biomaterials injected in mice pancreatic tumors up to 7-days post injection. Plot shows relaxation values corresponding to gadolinium contrast remaining in the tumor microenvironment for up to 7-days post injection.

MR/CT images of mice were taken prior to biomaterial injection and at designated time points post injection. MR imaging was used as a modality to investigate the imaging contrast provided by the LIFE biomaterial *in-vivo* (**Figure 2**). Different formulations of LIFE biomaterial were investigated to find the optimal one that will allow sustained visualization over-time sufficient for hypo-fractionated radiotherapy schedules. The combination of the sodium alginate with chitosan showed sustained MR contrast.

**Figure 3:**
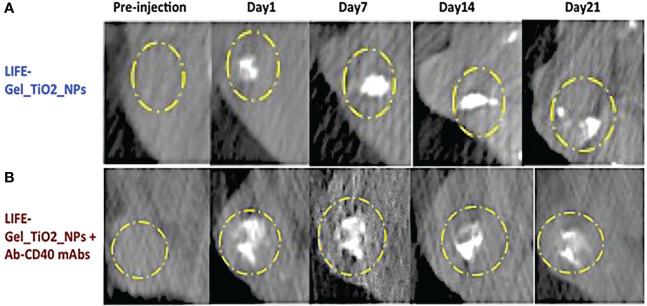
CT images showing contrast from LIFE biomaterials injected in mice pancreatic tumors. **(A)** The LIFE biomaterial formulation was prepared using a mixture of 4% (w/v) (v = 50 uL) alginate biomaterial solution added to a 1% (w/v) (v = 50 uL) chitosan solution in a 1:1 ratio. 10 uL volume titanium dioxide (TiO2) nanoparticles was added to the polymer mixture. **(B)** The LIFE biomaterial formulation was prepared as in **(A)** plus monoclonal antibody (mAb), Ab-CD40 (v = 2.3uL/injection), Study in mice was conducted to assess CT image contrast for up to 21-days.

Titanium dioxide nanoparticles were used as CT contrast agents to incorporate in the LIFE biomaterial to investigate its retention of the CT signal over time (**Figure 3**).

### 3.2 Efficacy of LIFE biomaterial loaded with anti-CD40 mAbs in pancreatic cancer.

**Figure 4:**
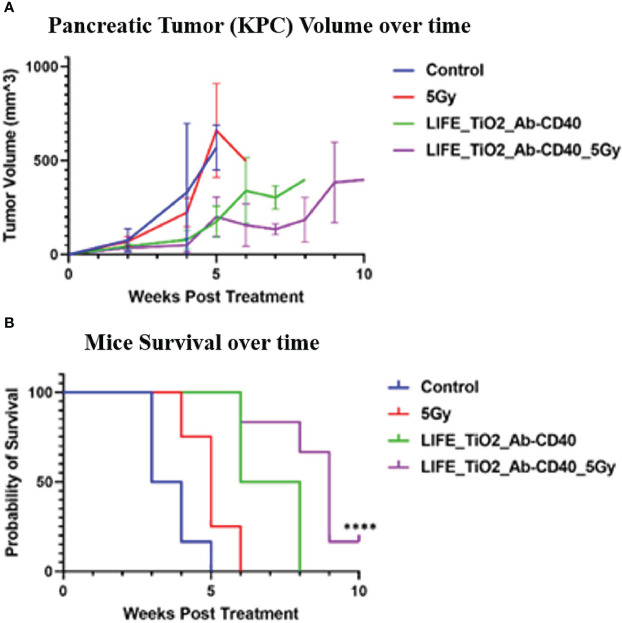
Mice tumor volume **(A)** and survival **(B)** were assessed for up to 10-weeks post treatment. Treatment was administered when tumor diameter reached ~4mm. The cohorts of mice investigated were: 1) control (n =6) received no treatment, 2) Radiotherapy dose of 5Gy, 3) mice treated with LIFE_TIO_2_ biomaterial loaded with 20ug of Ab-CD40, and 4) mice treated with LIFE_TIO_2_ biomaterial loaded with 20ug of Ab-CD40 exposed to 5Gy of radiation. Slowed tumor growth was observed for cohorts treated with LIFE_TiO_2__Ab-CD40 with/without 5Gy. However, mice survival appeared most favorable for cohort treated with LIFE_TiO_2__Ab-CD40_5Gy compared to control as indicated by the symbol '****' corresponding to p-value < 0.0001.

LIFE biomaterial was formulated with TiO_2_ nanoparticles to incorporate anti-CD40 monoclonal antibody in the treatment of mouse pancreatic tumor when average tumor diameter size reached ~4mm. Study endpoint was determined by tumor burden (size = 2 cm) or high degree of ulceration as established by protocol approved by the animal care and use committee. **Figure 4** displayed mice tumor volume assessment and their survival over time showing slowed tumor growth and longer overall survival for mice treated with LIFE_TiO_2_ and Ab-CD40.

## 4 Discussion

The results demonstrate potential for developing a new generation of liquid fiducial markers that can provide image-guidance during IGRT but also enhance therapy outcomes. The results provide a useful reference for more studies to optimize such formulations in more preclinical studies for clinical translation, including optimizing concentrations of nanoparticles and immunoadjuvants. The combination of chitosan and alginate polymers increase cross linkages for denser structure (19) (20). Other benefits of using sodium alginate and chitosan polymers are due to their biocompatibility, non-toxicity, low cost, and a simple transition into a functional biomaterial among other profits (21, 22).

The approach to incorporate nanoparticles optimized for IGRT and sustained delivery of immunoadjuvants represents a promising direction for innovation including towards making the tumor microenvironment more immunogenic. LIFE radiotherapy biomaterials incorporating immunoadjuvants proffer a highly customizable payload delivery system that may be able to combine radiotherapy with immunotherapy for boosting therapeutic outcome while minimizing toxicity. The incorporation of anti-CD40 in the LIFE biomaterial is expected to enhance therapy outcomes due to the additional therapeutic action of anti-CD40 (23). Among the different drug delivery systems designed for controlled delivery of payload (1, 24), the LIFE biomaterial presents a system that can serve as a fiducial and also include radio-sensitizing nanoparticles.

In **Figures 4A, B**, it can be observed that there is a modest difference when using the LIFE biomaterial with and without radiotherapy. Our analysis with two-tailed test shows no statistically significant difference (p-value = 0.183) between these two arms at 6-weeks post treatment. Altogether, this suggest that the effect of the LIFE biomaterial alone loaded with the anti-CD40 immunotherapy also has a therapeutic benefit as would be expected with anti-CD40. The advantage of using RT is that it can generate more neoantigens, broadening the repertoire of responding T cells, and enhancing efficacy as highlighted in previous studies (14, 23). The use of other doses of RT may provide a clearer additional benefit, with the results here justifying further investigation. The clear finding here consistent with the focus of this study is that the LIFE biomaterial used in this study can provide image-contrast for RT while also providing a therapeutic benefit. This is valuable in considering the LIFE biomaterial in developing a new generation of multifunctional fiducial markers.

Limitations of the current study include the limited number of animals used per cohort, and the lack of variations in radiation dose delivered to the tumor, as well as concentrations of the nanoparticles and immunoadjuvants. However, the study highlights the potential of such LIFE biomaterials loaded with different types of nanoparticles. Another limitation is that this study is conducted in a subcutaneous tumor model which does not necessarily reflect the complications and intricacies of pancreatic tumors. However, future studies will definitely explore the orthotopic tumor model for pancreatic cancer. These results motivate further studies varying these different parameters including using different high-Z nanoparticles and different types of immunotherapies to investigate their theragnostic capabilities for different types of solid tumors.

LIFE biomaterial may be most useful for hypo-fractionated radiotherapy or shorter treatment regimens since it biodegrades there is generally more compelling interest in implanting fiducial markers to reduce target margins and reduce exposure of adjacent organs at risk. There is an increasing trend towards increased adoption of hypo-fractionated radiotherapy especially when combining with immunotherapy to minimize the effect of radiotherapy on immune cells in addition to improving patient’s overall survival (25, 26). Many studies show hypo-fractionation can significantly enhance patient convenience, increase access to treatment and address disparities, with potential to significantly reduce treatment time and costs (27–29). The ability to easily administer the LIFE biomaterial could also be a significant benefit in resource-poor settings. The translational potential of this study is seen where the LIFE biomaterial can be employed in place of currently used fidicial markers, typically administered *via* endoscopic ultrasound-guided placement (30). This would come at no additional inconvenience to some patients undergoing image-guided radiotherapy with fiducials. Nevertheless, if the therapeutic outcome of increased survival can be optimized this can provide a viable treatment option for clinical translation, especially given the currently limited treatment options for pancreatic cancer.

In conclusion, this study shows that the LIFE biomaterial could be developed into a multi-functional fiducial that can provide image-guidance and enhance therapy outcomes. The LIFE biomaterial can be optimized for hypo-fractionated radiotherapy and combination with immunotherapy building on the inherent advantages of liquid fiducials.

## Data availability statement

The original contributions presented in the study are included in the article/supplementary material. Further inquiries can be directed to the corresponding authors.

## Ethics statement

The animal study was reviewed and approved by Institutional Animal Care and Use Committee (IACUC) of the Dana Farber Cancer Institute.

## Author contributions

GR helped to generate some of the data used in this study and reviewed the manuscript. SY-K helped in generating some of these data and reviewed the manuscript. AN and CD reviewed the manuscript and provided clinical perspective. WN is the principal investigator who designed the study, made substantial edits to the review of this manuscript. MM generated all the results in this study and designed the LIFE biomaterial for injection in mice and wrote the manuscript. All authors contributed to the article and approved the submitted version.
